# Editorial

**DOI:** 10.1120/jacmp.v3i1.2585

**Published:** 2002-01-01

I am very pleased to announce that with this first issue of the third volume of the Journal of Applied Clinical Medical Physics (JACMP), Karl Prado is joining the Editorial Board as an Associate Editor. He joins John Horton and Mike Herman as an Associate Editor in Radiation Oncology Physics. Both Associate Editors are doing a superb job, but with the increasing number of submissions in radiation oncology physics papers, further help is needed. John Horton will continue to take the prime responsibility for brachytherapy manuscripts, and Karl Prado will help Mike Herman with the external beam papers.

Except for the editorial office staff (i.e., the Editor‐in‐Chief and the Manuscript Manager), the Editorial Board volunteers their time and serves without compensation. Take a moment next time you see them or correspond with them to thank them for all the hard work that they put in on behalf of the journal. It would not be successful without them.

As an electronic journal we have the option of doing things not possible with a print journal; one of these being the use of short video sequences or loops as part of a manuscript. The JACMP is interested in initiating this option and we will be working on it during the year.

Peter R. Almond, Ph.D.

Editor‐in‐Chief

